# P087 Tongue necrosis as the presenting feature of giant cell arteritis

**DOI:** 10.1093/rap/rkag113.117

**Published:** 2026-11-03

**Authors:** Najla Elndari, Hassan Butt, Rihards Buss

**Affiliations:** Newcastle Hospitals NHS Foundation Trust, Newcastle upon Tyne, United Kingdom; Newcastle University, Newcastle upon Tyne, United Kingdom; Newcastle Hospitals NHS Foundation Trust, Newcastle upon Tyne, United Kingdom; Newcastle Hospitals NHS Foundation Trust, Newcastle upon Tyne, United Kingdom

## Abstract

**Introduction:**

Tongue necrosis is a rare ischaemic complication of giant cell arteritis (GCA), reported only in isolated cases and small series. Because it may mimic infection, malignancy or local trauma, affected patients frequently present to oral and maxillofacial or otolaryngology services rather than to rheumatology, and diagnosis is often delayed until tissue loss is irreversible. We report an older woman in whom GCA presented as necrotising glossitis with candidal colonisation or superadded infection and progressive trismus. Temporal artery ultrasound supported the diagnosis after tongue biopsy and cross-sectional imaging had excluded malignancy but had not demonstrated vasculitis.

**Case description:**

A 79-year-old woman was admitted under oral and maxillofacial surgery with painful glossitis and dysphagia. She described a four-month history of malaise, neck and occipital pain, and left temporal pain radiating to the shoulder, with progressively restricted mouth opening that had impaired her steroid inhaler use. Tongue pain had developed ten days before admission. There were no visual disturbances, limb claudication or proximal girdle symptoms. Inflammatory markers were markedly elevated: C-reactive protein (CRP) 312 mg/L, erythrocyte sedimentation rate (ESR) 105 mm/hr and platelets 580.

CT and MRI imaging of the neck demonstrated focal left hemitongue oedema without evidence of malignancy or collection. A tongue swab cultured Candida parapsilosis, and intravenous co-amoxiclav and fluconazole were commenced, with partial biochemical response. The anterior tongue subsequently developed established necrosis. Biopsy demonstrated inflammatory ulceration and tissue necrosis, without dysplasia, malignancy, granulomatous inflammation, vasculitis or fungal hyphae.

Rheumatology review identified a history consistent with GCA. Temporal artery ultrasound showed global wall thickening with absent flow throughout the left temporal artery tree and left facial artery, and flow preserved only in the right parietal branch. The lingual arteries could not be interrogated, as the patient was unable to protrude her tongue. CT and MR angiography of the aorta and its principal branches showed no features of large vessel vasculitis.

She received three days of intravenous methylprednisolone, followed by dispersible prednisolone 60 mg daily, as she was unable to swallow tablets. The necrotic anterior tongue autoamputated during toothbrushing, and residual slough was surgically excised, with viable tissue evident beneath. Speech and language therapy supported swallowing rehabilitation. Steroid-induced hypertension required treatment, and DEXA confirmed osteoporosis (spine T-score -3.4), for which she received zoledronic acid and colecalciferol and calcium supplements. She remains under rheumatology follow-up on a gradual prednisolone tapering regimen, with no recurrence of GCA symptoms.

**Discussion:**

GCA may present entirely outside rheumatology. In this patient the initial presentation was attributed to infective glossitis, and the isolation of Candida with partial response to antimicrobials was potentially misleading. The candidal infection most likely represented opportunistic colonisation of devitalised tissue, possibly compounded by poor inhaled corticosteroid technique secondary to restricted mouth opening. A partial antimicrobial response did not account for the overall clinical picture and, in retrospect, warranted earlier reassessment.

The preceding trismus is an important clinical clue. Progressive restriction of mouth opening may reflect masticatory ischaemia, analogous to jaw claudication, and in this case preceded tongue necrosis by several weeks. Earlier recognition may have allowed treatment before irreversible tissue loss.

The diagnostic value of tongue biopsy in the context of necrosis is limited. Lingual ischaemia in GCA arises from the lingual artery, proximal to the biopsied mucosa, such that superficial biopsy typically demonstrates only necrosis and secondary inflammation. Normal tongue histology therefore does not exclude the diagnosis. Temporal artery ultrasound established cranial arteritis in the temporal and facial arteries, which was sufficient to diagnose GCA and explain the lingual ischaemia.

Several management questions merit discussion. Glucocorticoids control systemic inflammation and reduce the risk of further ischaemic events, including visual loss, but established necrosis is irreversible; conservative management permitted demarcation and autoamputation with viable tissue beneath, and surgical excision was confined to residual slough. The role of early adjunctive interleukin-6 blockade in ischaemic presentations, and the optimal timing of surgical intervention for lingual necrosis, remain uncertain. The case also emphasises the importance of assessing bone protection at the outset of high-dose glucocorticoid therapy in older patients, in whom osteoporosis may be unrecognised.

**Key learning points:**

• GCA should be considered in any older patient presenting with tongue pain, ulceration or necrosis, particularly in the presence of headache, constitutional symptoms and raised inflammatory markers, even when infection or malignancy initially appears more likely.

• Progressive trismus may represent masticatory ischaemia and should prompt the same diagnostic urgency as jaw claudication.

• Tongue biopsy in lingual ischaemia typically demonstrates only necrosis and non-specific inflammation, as the arteritis affects vessels proximal to the biopsy site.

• Superadded infection or colonisation of necrotic tissue can obscure the underlying diagnosis. Partial response to antimicrobials should not provide false reassurance when the overall clinical picture suggests ischaemia.

• The diagnosis of GCA is clinical, integrating history, examination, inflammatory markers and imaging. Temporal artery ultrasound contributed decisively here and retained value even though the symptomatic lingual vessels could not be directly interrogated.

• Established necrosis is irreversible; however, prompt glucocorticoid therapy protects against further ischaemic complications, and conservative management of lingual necrosis may permit demarcation and autoamputation with preservation of underlying viable tissue. The role of early adjunctive tocilizumab in severe ischaemic cranial GCA remains an important area for discussion.

• Bone protection should be assessed at the outset of high-dose glucocorticoid therapy in older patients.

